# Systematic Evaluation of Candidate Blood Markers for Detecting Ovarian Cancer

**DOI:** 10.1371/journal.pone.0002633

**Published:** 2008-07-09

**Authors:** Chana Palmer, Xiaobo Duan, Sarah Hawley, Nathalie Scholler, Jason D. Thorpe, Rob A. Sahota, May Q. Wong, Andrew Wray, Lindsay A. Bergan, Charles W. Drescher, Martin W. McIntosh, Patrick O. Brown, Brad H. Nelson, Nicole Urban

**Affiliations:** 1 Canary Foundation, Scientific Programs, San Jose, California, United States of America; 2 British Columbia Cancer Agency, Trev & Joyce Deeley Research Center, Victoria, Canada; 3 Department of Gynecology and Obstetrics, University of Pennsylvania School of Medicine, Philadelphia, Pennsylvania, United States of America; 4 Fred Hutchinson Cancer Research Center, Division of Public Health Sciences, Seattle, Washington, United States of America; 5 Department of Biochemistry, Stanford University, Stanford, California, United States of America; 6 Howard Hughes Medical Institute, Stanford, California, United States of America; Dalhousie University, Canada

## Abstract

**Background:**

Epithelial ovarian cancer is a significant cause of mortality both in the United States and worldwide, due largely to the high proportion of cases that present at a late stage, when survival is extremely poor. Early detection of epithelial ovarian cancer, and of the serous subtype in particular, is a promising strategy for saving lives. The low prevalence of ovarian cancer makes the development of an adequately sensitive and specific test based on blood markers very challenging. We evaluated the performance of a set of candidate blood markers and combinations of these markers in detecting serous ovarian cancer.

**Methods and Findings:**

We selected 14 candidate blood markers of serous ovarian cancer for which assays were available to measure their levels in serum or plasma, based on our analysis of global gene expression data and on literature searches. We evaluated the performance of these candidate markers individually and in combination by measuring them in overlapping sets of serum (or plasma) samples from women with clinically detectable ovarian cancer and women without ovarian cancer. Based on sensitivity at high specificity, we determined that 4 of the 14 candidate markers-MUC16, WFDC2, MSLN and MMP7-warrant further evaluation in precious serum specimens collected months to years prior to clinical diagnosis to assess their utility in early detection. We also reported differences in the performance of these candidate blood markers across histological types of epithelial ovarian cancer.

**Conclusions:**

By systematically analyzing the performance of candidate blood markers of ovarian cancer in distinguishing women with clinically apparent ovarian cancer from women without ovarian cancer, we identified a set of serum markers with adequate performance to warrant testing for their ability to identify ovarian cancer months to years prior to clinical diagnosis. We argued for the importance of sensitivity at high specificity and of magnitude of difference in marker levels between cases and controls as performance metrics and demonstrated the importance of stratifying analyses by histological type of ovarian cancer. Also, we discussed the limitations of studies (like this one) that use samples obtained from symptomatic women to assess potential utility in detection of disease months to years prior to clinical detection.

## Introduction

Epithelial ovarian cancer (EOC) has the highest mortality of all gynecological cancers and is the fifth leading cause of cancer death among women in the United States. In 2007, there were 22,430 new cases of EOC and an estimated 15,280 deaths in the United States [1]. The five year survival rate for EOC in the US is approximately 45%, due largely to the high proportion of EOCs that are not detected until they have spread outside the ovary [2]. There are four major histological types of EOC: serous, endometrioid, clear cell, and mucinous. These four histological types are enormously different, in both clinical and molecular characteristics. The serous subtype is the most commonly diagnosed and is responsible for most ovarian cancer deaths [2].

Early detection is a promising approach to reducing mortality from cancers that are most often diagnosed in their late stages [3]. Because the histological types of ovarian cancer are intrinsically different diseases, the optimal strategies for early detection, and the cost-benefit calculations in evaluating their performance, may be different for each subtype. The potential benefit of early detection is greatest for serous EOC because it is the most common and lethal ovarian cancer subtype, and it has therefore been the primary target of our efforts.

The clinical utility of a diagnostic test is often expressed in terms of positive predictive value (PPV)–the fraction of test positives that are true positives. To be justified for clinical use, a diagnostic test must achieve a PPV that balances the benefits of early detection against the cost of the test and risk associated with false positives (e.g. anxiety, unnecessary surgery). A PPV of at least 10%, meaning that 10% of women that test positive actually have the disease, has often been used as a somewhat arbitrary target for an early detection test for ovarian cancer [4]. A major factor in the challenging nature of early detection of serous EOC is the low incidence of the disease in the general population, which implies that a screening test must be highly specific in order to avoid over-diagnosis and over-treatment. In the general population, to achieve a PPV of 10%, the performance requirements are extremely high: given the age-adjusted annual incidence rate of all EOC in women over age 50 in the US of 35 per 100,000 [5], a test must achieve 99.7% specificity at 80% sensitivity. The specificity required for selective detection of the serous subset of EOC in the general population (which has a lower incidence than the figure above) would be correspondingly higher. In order to achieve a PPV of 10% for detecting serous EOC among BRCA1 mutation carriers, a test must achieve a specificity requirement of 78.1% at 80% sensitivity given the incidence of serous ovarian cancer over age 50 in this population is approximately 3000 cases per 100,000 [6]. One must bear in mind, however, that this performance could be achieved through the combined performance of a blood test as a first-line screen and follow-up imaging test. Furthermore, the threshold for an acceptable PPV depends on the intervention and it may be that a PPV less than 10% could be acceptable.

The best-studied serum marker for ovarian cancer, CA125 (MUC16), has been evaluated extensively for its utility as a marker of ovarian cancer, and is FDA approved for recurrence monitoring. In retrospective studies, CA125 has been shown to signal disease recurrence roughly six months prior to the development of symptoms [7]. In women with clinically detected stage I EOC (of various histologies), pre-operative serum levels of CA125 are elevated (>35 U/ml) in roughly 66% of women [8]. In the Janus longitudinal cohort, CA125 has been shown to contain potential signals in the blood as early as five years before clinical detection [9], and to have an estimated sensitivity of 45% at 93% specificity at 1.5 years prior to diagnosis among women over 50 years of age, which is encouraging but far from adequate for clinical use [10].

These results provide an important example of the difference in marker performance in clinically diagnosed disease versus pre-symptomatic disease–the true target of an early detection test. The reduction in performance from clinically diagnosed tumors (even Stage I) to pre-symptomatic disease is not surprising given that clinically diagnosed cancers are almost certainly in general much larger than the early tumors we need to detect to improve survival, and underscores the importance of evaluating candidate markers in specimens from pre-symptomatic women. Unfortunately, due to limitations in specimen availability, most studies of marker performance (including this one) have evaluated performance in clinical samples collected from women who already have signs and symptoms of cancer.

In recent years, the application of genomic and proteomic technologies has fueled an explosion in marker discovery efforts in various diseases, including EOC. Some studies have evaluated combinations of two or more markers in order to identify the sets that work best together in a panel. Such studies are essential because it is unlikely that any single marker will have adequate performance in detecting cancers prior to the development of symptoms. While evaluation of a candidate marker's contribution to a panel in specimens from women with clinically apparent ovarian cancer may be a poor predictor of its lead time and utility in early detection, it provides a useful filter for gaining access to precious pre-clinical specimens.

We undertook a systematic performance evaluation of 14 candidate blood-based markers for EOC selected based on a gene expression data and published literature. Our candidate marker list included: MUC16 (CA125), WFDC2 (HE4), MSLN, IGF2, CHI3L1 (YKL40), MMP7, MIF, PRL, SPP1 (OPN), BMP7, LCN2, IL13RA2, TACSTD1 (EpCam), and AMH. Note that all markers were referred to by their HUGO gene symbols. We evaluated these markers using common sets of well annotated EOC cases and control serum samples, including women with healthy ovaries as well as women with benign and malignant ovarian conditions. Our objective was to use performance in these clinically diagnosed cases as a filter to assess which candidate markers warranted further evaluation in precious serum specimens obtained months to years prior to diagnosis of ovarian cancer. We also used these data to conduct analyses of marker panels (a named group of markers) and composite markers (which include a specific classification or combination rule) as well as to explore the effect of stratifying analyses by histological type.

## Results

### Marker Selection

We selected candidate markers by using gene expression data to identify genes highly expressed in ovarian cancer but not in the rest of the body, as described in Materials and Methods. Using this strategy, the following candidate markers with commercially available ELISAs or other published assays were selected for testing: MSLN, WFDC2, IGF2, CHI3L1, MMP7, BMP7, LCN2, TACSTD1. Many of these markers have previously been reported to be elevated in women with ovarian cancer [11]–[22]. Several other candidate markers were also tested based on literature and/or collaborative opportunities: MUC16, IL13RA2, PRL, MIF, SPP1 and AMH [8], [23]–[25].

### Evaluation of individual markers

In order to optimize analysis of marker combinations, we evaluated each candidate marker in common sets of well annotated EOC cases and control serum samples, including women with healthy ovaries, as well as women with benign and malignant ovarian conditions. The 14 candidate markers for EOC described above were evaluated in a stepwise manner using three overlapping serum sets of increasing complexity: the Filtering set, Mini-Triage set, and Triage set (Table 1). The composition of each of the serum sets with regard to stage and tumor histology is described in Table 2.

**Table 1:** Marker evaluation pipeline: cases and controls.

Patient Class	Filtering Set	Mini-Triage Set	Triage Set	Overlap^a^
Ovarian Cancer Cases	50	35	71	17
Healthy Controls^b^	9	12	58	12
Surgical Benigns^c^	0	16	53	16
Surgical Normals^d^	0	8	32	8
**Total**	**59**	**71**	**214**	

a) Overlapping specimens in the Mini-Triage and Triage sets

b) Healthy Controls: women enrolled in prospective screening trials who remained free of ovarian cancer for at least two years after serum collection.

c) Surgical Benigns: women with surgically confirmed benign ovarian pathology.

d) Surgical Normals: women that underwent surgery but no ovarian pathology was identified.

**Table 2:** Stage and histology of ovarian cases in each serum set.

**Filtering Set**						
**Stage**	**Serous**	**Clear Cell**	**Endometrioid**	**Mucinous**	**Adeno.NOS** a	**Total**
**I**	0	0	0	0	0	**0**
**II**	0	0	0	0	0	**0**
**III**	30	2	2	0	3	**37**
**IV**	9	0	1	0	1	**11**
**None**	1	0	0	0	1	**2**
**Total**	**40**	**2**	**3**	**0**	**5**	**50**
Mini-Triage Set						
**Stage**	**Serous**	**Clear Cell**	**Endometrioid**	**Mucinous**	**Adeno.NOS** a	**Total**
**I**	3	2	2	0	1	**8**
**II**	1	0	2	0	1	**4**
**III**	11	2	1	1	4	**19**
**IV**	1	0	1	0	2	**4**
**Total**	**16**	**4**	**6**	**1**	**8**	**35**
Triage Set						
**Stage**	**Serous**	**Clear Cell**	**Endometrioid**	**Mucinous**	**Adeno.NOS** a	**Total**
**I**	7	5	2	5	4	**23**
**II**	2	0	2	0	0	**4**
**III**	32	0	1	1	5	**39**
**IV**	3	0	1	0	1	**5**
**Total**	**44**	**5**	**6**	**6**	**10**	**71**

a AdenoNOS = Adenocarcinoma Not Otherwise Specified

The first step of candidate marker evaluation was the Filtering set of sera, a series of mixtures in varying ratios of pooled EOC sera from EOC patients and control serum pools from volunteers who did not have EOC. This test served as a first cut to eliminate assays that did not show a consistent difference between cases and controls with minimal use of case and control specimens. This filter was essentially an endogenous standard curve; failure to show a linear relationship between case to control ratio and ELISA signal indicated either that marker levels were roughly the same in most cases as in controls or that the assay was not sensitive enough to detect a small increase (or decrease) in marker levels in cases. Eight of the 11 candidate markers tested in the filtering set showed a linear relationship between the ratio of EOC patient serum to control serum in the samples and the signal measured in the corresponding ELISA assay, while three of the candidate markers (TACSTD1, AMH, IL13RA2) showed no consistent relationship between these values and were not evaluated further.

**Figure 1:**
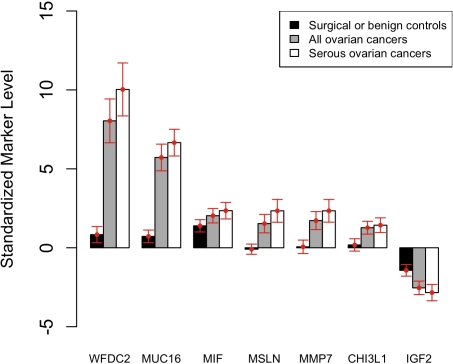
Normalized serum marker levels. Bar heights indicate the mean of the normalized values of a given marker in the specified subset of cases or controls. Error bars represent the 95% confidence interval associated with the mean. The logarithm of serum marker concentrations were normalized to standard deviations from the mean of the corresponding measurements in Healthy Controls.

The eight markers that passed analysis in the Filtering set, as well as two previously validated EOC markers (MUC16 and MSLN [17], [26]), were further tested in the Mini-Triage serum set (n = 71). Four markers (PRL, SPP1, BMP7, LCN2) showed poor performance (sensitivity <10% at 98% specificity and area under curve (AUC)<0.70) in the Mini-Triage set and were not pursued further. The remaining six candidate markers, as well as previously validated EOC marker WFDC2 [12], [27], were tested in the larger Triage serum set (n = 214). Markers tested on this expanded dataset were assessed by a number of criteria, including sensitivity at 98% specificity, AUC, and mean normalized serum marker levels in specific subsets of cases and controls (Table 3). The known markers MUC16, WFDC2, and MSLN showed the best performance according to 98% specificity for all cases versus all controls, with sensitivities of 70%, 61%, and 30%, respectively. These three markers also showed the best performance when only cases of serous histology were considered (sensitivities at 98% specificity of 86%, 75%, and 45%, respectively). For each marker, we also calculated the distance between EOC patients and Healthy Controls (women enrolled in prospective screening trials who remained free of ovarian cancer for at least two years after serum collection.) by normalizing all measurements to the mean levels in these healthy specimens (see Statistical Methods). With this normalization, the mean level among the case group represented a “discriminatory distance” measure that reflected how far the average case was from the average healthy control. MUC16 and WFDC2 were clearly superior according to this metric, with mean normalized serum marker concentrations in cases (relative to Healthy Controls) of 6.7 and 10.0, respectively. By comparison, the other markers all scored less than 2.5 (Figure 1 and Table 3).

**Table 3:** Single marker performance.

Serum Set	Mini-Triage (35 Cases, 36 Controls)			Triage (71 Cases, 143 Controls)							
		
Cases	All Cases (N = 35)			All Cases (N = 71)				Serous Cases (N = 44)			
			
Gene Symbol	Sens98^a^	Sens95^b^	AUC^c^	Sens98^a^	Sens95^b^	AUC^c^	Discriminatory Dist. Mean, SE (95% CI)^d^	Sens98^a^	Sens95^b^	AUC^c^	Discriminatory Dist. Mean, SE (95% CI)^d^
MUC16	60%	86%	0.95	70%	82%	0.92	5.72, 0.42 (4.87, 6.57)	86%	93%	0.98	6.66, 0.42 (5.81, 7.51)
WFDC2	N/A	N/A	N/A	61%	63%	0.90	8.04, 0.70 (6.65, 9.43)	75%	77%	0.96	10.03, 0.84 (8.35, 11.71)
MSLN	31%	31%	0.66	30%	39%	0.73	1.53, 0.30 (0.95, 2.11)	45%	55%	0.85	2.34, 0.36 (1.62, 3.06)
IGF2	17%	26%	0.83	18%	31%	0.80	−2.54, 0.22 (−2.97, −2.11)	25%	41%	0.84	−2.84, 0.26 (−3.36, −2.32)
CHI3L1	9%	37%	0.83	10%	31%	0.75	1.27, 0.21 (0.86, 1.68)	11%	34%	0.77	1.43, 0.23 (0.96, 1.90)
MMP7	40%	51%	0.80	21%	35%	0.74	1.72, 0.29 (1.15, 2.29)	32%	48%	0.81	2.34, 0.35 (1.62, 3.06)
MIF	17%	34%	0.79	10%	20%	0.72	2.03, 0.23 (1.57, 2.49)	11%	20%	0.77	2.35, 0.26 (1.83, 2.87)
PRL	0%	0%	0.65								
SPP1	0%	3%	0.40								
BMP7	3%	6%	0.42								
LCN2	6%	20%	0.63								

a) Sens98 = Sensitivity at 98% specificity in discriminating cases from all controls (Healthy Controls, Surgical Benigns and Surgical Normals).

b) Sens95 = Sensitivity at 95% specificity in discriminating cases from all controls (Healthy Controls, Surgical Benigns and Surgical Normals).

c) AUC = Area Under (ROC) Curve for discriminating cases from all controls (Healthy Controls, Surgical Benigns and Surgical Normals).

d) Discriminatory Dist. = Discriminatory Distance. The logarithm of serum marker concentrations were normalized to standard deviations from the mean of the corresponding measurements in Healthy Controls. Mean, SE (95% CI) = Mean, standard error, and 95% confidence intervals of difference between normalized serum marker concentration in cases and in Healthy Controls.

### Evaluation of combination markers

For the seven markers evaluated in the Triage set, we tested all markers in combinations of either two or three, and compared individuals without cancer to all ovarian cancer patients or serous cases only. The best-performing two-marker combination in either analysis was MUC16 and WFDC2 (Table 4). This two-marker combination yielded sensitivities at 98% specificity of 72% for all cases and 86% for serous cases only, compared to sensitivities at 98% specificity for MUC16 alone of 70% (all cases) and 86% (serous cases). However, the performance of the best two marker combination (MUC16 and WFDC2) was not significantly different from the best single marker (MUC16) as measured by pAUC (from specificity = 100% to specificity = 90%) between the best single marker and the best two marker combination. Evaluation of all potential three-marker combinations (data not shown) also failed to show any improvement in performance in either sensitivity at 98% specificity or AUC.

**Table 4:** Combination marker performance.

	All Cases (N = 71)			Serous Cases (N = 44)		
Gene Symbol	Sens98^a^	AUC^b^	P-value^c^	Sens98^a^	AUC^b^	P-value^c^
MUC16	70%	0.92	N/A	86%	0.98	N/A
MUC16+WFDC2	72%	0.92	0.342	86%	0.99	0.187
MUC16+WFDC2+MSLN	72%	0.91	1.000	86%	0.99	1.000
MUC16+WFDC2+MIF	72%	0.93	0.396	86%	0.99	1.000
MUC16+WFDC2+IGF2	72%	0.94	0.353	86%	0.99	1.000
MUC16+WFDC2+MMP7	72%	0.91	0.748	86%	0.99	1.000
MUC16+WFDC2+CHI3L1	72%	0.92	1.000	86%	0.99	1.000

a) Sens98 = Sensitivity at 98% specificity in discriminating cases from all controls (Healthy Controls, Surgical Benigns and Surgical Normals).

b) AUC = Area Under (ROC) Curve for discriminating cases from all controls (Healthy Controls, Surgical Benigns and Surgical Normals).

c) P-value for the best available two-marker combination compared to the best available three-marker combination (see Materials and Methods).

### Performance evaluation in the context of histological type and stage

To investigate how marker performance varied with histological type and stage, we calculated the number and percentage of cases correctly classified by the MUC16/WFDC2 combination marker by histology and stage, for all cases versus all controls (Healthy Controls, Surgical Benigns, and Surgical Normals–see Clinical Blood Specimens) in the Triage set using 98% specificity for this combination marker. The performance of the MUC16/WFDC2 combination marker clearly varied with ovarian cancer histology: the percentage of cases correctly classified was 86% (38/44) for serous cases, 83% (5/6) for endometrioid cases, 17% (1/6) for mucinous cases and 0% (0/5) for clear cell cases (Table 5). Other markers whether used alone, in pairs, or combinations of three, showed similarly poor performance in clear cell and mucinous cases (data not shown).

**Table 5:** Summary of correctly identified cases by histological type.

	Stage 1	Stage II	Stage III	Stage IV	Total
**Serous**	86% (6/7)	50% (1/2)	88% (28/32)	100% (3/3)	**86% (38/44)**
**Endometrioid**	50% (1/2)	100% (2/2)	100% (1/1)	100% (1/1)	**83% (5/6)**
**Mucinous**	20% (1/5)	N/A (0/0)	0% (0/1)	N/A (0/0)	**17% (1/6)**
**Clear Cell**	0% (0/5)	N/A (0/0)	N/A (0/0)	N/A (0/0)	**0% (0/5)**
**Other**	25% (1/4)	N/A (0/0)	100% (5/5)	100% (1/1)	**70% (7/10)**
**Total**	**39% (9/23)**	**75% (3/4)**	**87% (34/39)**	**100% (5/5)**	**72% (51/71)**

Marker performance also appeared to vary with stage–the MUC16/WFDC2 combination marker correctly identified 39% (9/23) of Stage I cases, 75% (3/4) of Stage II cases, 87% (34/39) of Stage III cases and 100% (5/5) of Stage IV. However, these results are confounded by the different mix of histologies across stages. In the Triage set, the majority of stage III and IV cases were serous cancers (35/44), while the majority of stage I and II cases were non-serous cancers (endometrioid, mucinous, clear cell and other) (18/27). This sample composition reflected the fact that serous cancers are more likely to be identified at a late stage, while the reverse is true for endometrioid, clear cell and mucinous cancers.

## Discussion

We evaluated 14 candidate early detection markers for EOC using three overlapping sets of serum samples collected from women with clinically diagnosed EOC and cancer-free women with and without gynecological diseases. Markers were evaluated in successively larger serum sets depending on their performance at the previous step. For markers that graduated to the largest sample set, we evaluated the performance of each marker individually and in combinations of two or three for their ability to detect clinically diagnosed ovarian cancer.

Three assays (TACSTD1, AMH, IL13RA2) failed to detect the corresponding marker in pooled sera from EOC cases or pooled control sera. These results suggest that if there is a difference between cases and controls for these markers it must be very small and we would need a more sensitive assay to detect the difference. Four markers: PRL, SPP1, BMP7 and LCN2, passed the Filtering set (pools of cases and controls) but had poor sensitivity and specificity when evaluated in individual case and control specimens. A further evaluation of PRL in a serum set containing more surgical controls revealed that the elevated serum PRL levels were related to the blood collection conditions rather than to disease state [28]. These observations underscore the importance of well-controlled and well-documented collection procedures, and careful selection of controls, as some groups continue to report that prolactin is a useful marker of ovarian cancer without adequate attention to the matching of controls [29].

We found that marker levels varied considerably among histological types and clinical stages of ovarian cancer. All of the markers and combinations in this analysis had higher sensitivity at 98% specificity when only the serous EOC cases were considered, as compared to all cases of EOC. This difference appeared to be due to the poor performance of our candidate markers in clear cell and mucinous cases. In terms of sensitivity at 98% specificity, the best performing markers for the detection of clinically apparent serous EOC were MUC16, WFDC2 and MSLN, with sensitivities at 98% specificity of 86%, 75%, and 45%, respectively, in the Triage set of serum samples. No combination of markers provided a significantly better sensitivity at 98% specificity than the best individual marker, MUC16, in distinguishing all ovarian cancers or serous ovarian cancers from controls. The high positive correlation (range: 0.54–0.75) among the three best performing single markers contributed to the lack of significant improvement in sensitivity when combining markers in this study.

Our data analysis approach differed from those in most previous studies in several significant ways. First, we stratified our results by histological type, with an emphasis on serous EOC. Ovarian cancers of different histological types are well known to have very different clinical and molecular characteristics, yet they are often erroneously grouped together in analyses of marker performance, presumably for the sake of greater sample size. We focused our analyses on the serous subtype of EOC because early detection of high-grade serous cancers has the greatest potential to save lives. This decision to stratify was supported by our finding that the markers examined here consistently performed better in serous and endometrioid cases than in clear cell and mucinous cases (even in Stage I cases only), and consequently that marker performance was lower in a pooled case set than in serous cases alone. Second, we elected not to stratify our analysis by stage of disease, as we are not confident that clinical Stage I/II high grade serous cancer is a useful model for the clinically occult precursors to lethal ovarian cancers which are the true targets of early detection. Furthermore, the distribution of histologies varied with stage, confounding interpretation of stage-specific results. Third, we chose to use unconventional measures of marker performance. The low prevalence of EOC requires a highly specific marker to reduce the risk of false positives in healthy women so as to avoid unnecessary distress, diagnostic follow-up and surgery. By contrast, the conventional AUC analysis indiscriminately summarizes the performance of a marker at all levels of specificity. Although we have included AUC values in this report, we consider the sensitivity of an assay at 98% specificity to be a more salient measure of its performance. We recognize, however, that even with superlative sensitivity, 98% specificity is still not sufficient for an early detection test in a normal-risk population.

Finally, we included a measure of magnitude of difference in signal between EOC patients and apparently healthy volunteers. We believe this metric is useful for helping to predict the value of a marker for early detection when using clinically detected cases because high signal at the time of symptoms may be consistent with discernible signal earlier in the course of the disease when tumor burden is lower and signal is presumably lower. MUC16 and WFDC2 were the only markers that showed large elevations in cases relative to Healthy Controls (6.7 and 10.0 discriminatory distance units, respectively). Markers having the same sensitivity and specificity can have very different discriminatory distance measures, and those with greater distance may be better candidates for early detection applications because they may maintain their performance better with smaller tumor burdens as marker levels attenuate toward control levels.

An important factor to consider in interpreting our results and other similarly designed studies is that these markers were evaluated based on their ability to distinguish between serum specimens from women with and without clinically apparent ovarian cancer. It is crucially important to keep in mind that the value of a marker for early detection is determined by its ability to detect ovarian cancer prior to development of clinical signs or symptoms (and, moreover, prior to progression to an advanced stage). Thus, until the performance of a candidate marker is evaluated with specimens from women with asymptomatic, early stage cancer, its value as an early detection marker remains hypothetical, and researchers must be cautious not to overstate their claims when assays have only been tested on samples from women with clinically detectable disease [29]. Furthermore, the relationship between marker performance in specimens collected at the time of diagnosis to performance during the window of opportunity for early detection is not well understood, and may vary considerably among markers.

The results presented here are encouraging, but much more work needs to be done before we will know whether we are in range of an effective early detection test for EOC. Specifically, it will be essential to evaluate markers in serum samples obtained prior to disease detection, in samples from women with clinically occult, localized serous cancers. Samples collected prior to disease diagnosis are a limited and precious resource, and samples from women with unsuspected, occult, localized cancers (e.g., discovered at risk reducing salpingo-oophorectomy) are even more precious, so careful selection of the markers worthy of evaluation in these samples is critical. Given the uncertain relationship between marker performance prior to diagnosis and performance at or after diagnosis of ovarian cancer, we believe that markers that demonstrate adequate performance individually but do not complement MUC16 in clinical (at-diagnosis) samples should not be excluded from further evaluation. We therefore intend to proceed with evaluation of MUC16, WFDC2, MSLN and MMP7, all of which have sensitivity >30% at 98% specificity in detection of clinically apparent serous cancers, beginning with analysis of serum specimens collected months to years prior to diagnosis of serous ovarian cancers.

Future work toward early detection of serous ovarian cancer may also benefit from expanded discovery efforts. Recent studies of the early natural history of EOC suggest that some cases of serous EOC may originate in the fallopian tubes (FT). In women with a germline mutation in BRCA1 or BRCA2, occult malignancy of serous histology, intraepithelial carcinoma or dysplasia is frequently found in the fimbrial end of the FT at the time of prophylactic surgery [30], [31]. In fact, prophylactic removal of fallopian tubes and ovaries in women at genetically high risk of EOC is a proven strategy for reducing mortality from ovarian cancer. In light of these findings, it may be useful to consider genes highly and specifically expressed in early stage serous fallopian tube cancers as potential markers of serous ‘ovarian’ cancer (whereas previous efforts focused on late stage ovarian tumors). In addition, advances in proteomic technologies have made it possible to do in-depth profiling of serum proteins, which, if applied to pre-diagnostic specimens could prove to be an effective means of identifying relevant markers. Ongoing efforts using targeted discovery, thoughtful combination of markers, and stratification of screening populations by cancer risk may yet lead to an effective early detection test for ovarian cancer.

## Materials and Methods

### Marker selection

The goal of our marker selection was to identify genes whose protein products are consistently found at higher levels in the blood of patients with early stage serous ovarian cancer than in healthy individuals. Our general strategy for achieving this goal was to identify genes that were highly expressed in serous ovarian cancers but minimally expressed in most normal tissues. We further favored genes that were known to encode secreted proteins. The gene expression data used to estimate gene expression in ovarian tumors included cDNA microarray profiles of 72 ovarian tumors, of which most were late stage tumors of serous histology (manuscript in preparation). Data on gene expression (as reflected by mRNA levels) in normal tissues were obtained from a published study of 115 human tissue samples representing 35 different tissue types, using cDNA microarrays representing approximately 26,000 different human genes [32]. Based on these criteria, the following candidate markers with available serum assays were selected for testing: WFDC2, MSLN, IGF2, CHI3L1, MMP7, BMP7, LCN2, TACSTD1. Several other markers were also tested based on literature and/or collaborative opportunities: MUC16, IL13RA2, PRL, MIF, SPP1 and AMH [8], [23]–[25].

### Clinical blood specimens

Study participants were recruited between June 1 1998 and July 1 2002 to support protocols of the Pacific Ovarian Cancer Research Consortium (POCRC) by physicians at Pacific Gynecology Specialists, Swedish Medical Center, Providence Medical Center, the University of Washington/Seattle Cancer Care Alliance, and Virginia Mason Medical Center. Cases were defined as having invasive epithelial carcinoma confirmed by standardized review of medical records and pathologist examination of paraffin-embedded tissue for tumor histology. FIGO stage and histology of the cases are summarized in Table 2. Blood was also obtained from three categories of controls: i) “Healthy controls”-apparently healthy women enrolled in prospective screening trials who remained free of ovarian cancer for at least two years after serum collection; ii) “Surgical Benigns”–women with surgically confirmed benign ovarian pathology ii) “Surgical Normals”–women that underwent surgery but no ovarian pathology was identified (Table 1). Each patient provided written informed consent and a medical records release form approved by the FHCRC institutional review board (IR file number #4771). Surgical specimens were obtained prior to any treatment or surgery (but after the administration of anesthesia). All specimens were anonymized for patient confidentiality.

Blood was drawn into three or four 10.0 ml SST (serum separator) Vacutainer blood collection tubes (Fisher Scientific Cat. # 02-683-98, Mfg. No.: 367985) as well as one lavender-top EDTA Vacutainer blood collection tube (Fisher Scientific Cat. # 02-657-32). Blood was processed and placed in the freezer within 4 hours of the collection time. All tubes were spun in a balanced centrifuged at 1,200×g for 10 minutes to separate serum from cellular components the cells from the fluid. Serum from the SST tubes and plasma from the EDTA tube were aliquoted into microcentrifuge tubes at 1 ml per aliquot and stored at −80°C. All markers were evaluated with serum with the exception of SPP1 (osteopontin) which was evaluated using EDTA plasma as per manufacturer's instructions (see Table 6).

**Table 6:** Commercial ELISA reagents.

Gene Symbol	Alias	Name	Assay Source^a^	Sensitivity (ng/ml)
AMH	MIS	anti-Mullerian hormone	DSL	0.01
BMP7		bone morphogenetic protein 7	RayBio	20
CHI3L1	YKL-40	chitinase 3-like 1	Quidel	1.67 (U/ml)
IGF2		insulin-like growth factor 2	DSL	0.002–0.058
IL13RA2		interleukin 13 receptor, alpha 2	Anogen	0.0005–0.004
LCN2	MMP-9; NGAL	lipocalin 2	R&D Systems	2.2
LCN2	MMP-9; NGAL	lipocalin 2	Ab Shop	0.11
MIF		macrophage migration inhibitory factor	Onco Detectors	0.14
MMP7		matrix metallopeptidase 7	R&D Systems	0.1
PRL		Prolactin	DSL	0.016
SPP1	Osteopontin	secreted phosphoprotein 1	Assay Designs	10
TACSTD1	Ep-CAM	tumor-associated calcium signal transducer 1	BioVendor	0.017

a) All assays were conducted on serum with the exception of SPP1 which was conducted using plasma-EDTA. See Table S2 for catalogue numbers.

Markers were evaluated using three overlapping sets of blood specimens, detailed in Table 1. (1) The Filtering set comprised a series of mixtures of two pools of serum samples from (a) 50 late stage EOC patients and (b) 9 age-matched apparently healthy women. The case and control sera were serially diluted to create a series of samples with defined ratios (fraction of case pool/total = 1/1, 1/2, 1/4, 1/8, 1/16, 1/32, 1/64, 1/128) of case and control pooled patient serum. We used the Filtering set to test for a difference in marker levels between case and control pools as measured by a linear relationship between the relative ratio of cases to controls and the immunoassay signal. Prior to testing in the filtering set, assays were evaluated with manufacturer supplied standard curves to assess limits of detection. (2) The Mini-Triage set comprised serum samples from 71 women, including patients with different histological types and stages of EOC, women with benign ovarian tumors (Surgical Benigns), women with healthy ovaries undergoing surgery for other gynecologic conditions (Surgical Normals), and age-matched women enrolled in prospective screening trials who remained free of ovarian cancer for at least two years after serum collection (Healthy Controls). This set of samples provided a preliminary estimate of the specificity and sensitivity of each immunoassay. (3) The Triage set consisted of an expanded set of 214 serum samples including specimens from 71 EOC patients (various histologies), and greater numbers of Healthy Controls, Surgical Benigns, and Surgical Normals. The Triage set had 17 cases and 36 controls in common with the Mini-Triage set. The Triage set was selected subsequent to the Mini-Triage, as some specimens did not have sufficient volume remaining for further testing after being used in the Mini-Triage set. Specimen aliquots used in the Triage and Mini-Triage sets were delivered to laboratories separately in a blinded fashion and were tested independently. The Triage set served as an in-depth verification of results in the from the Mini-Triage set. Table 1 describes the case/control composition of each serum set while Table 2 describes the breakdown of cases by stage and histology for each set.

### Immunoassays

Serum levels of MUC16, WFDC2 and MSLN were measured using bead-based immunoassay consistent with previously published methods [27], [28], [33] (Table 7, Table S1). The mAbs were dialyzed against Dulbecco's phosphate buffered saline (PBS) (Invitrogen Corporation, Carlsbad, CA) when necessary. Detection antibodies were biotinylated using the EZ-Link-sulfo-NHS-biotinylation kit (Pierce, Rockford, IL) and dialyzed (G Biosciences Tube-O-Dialyzer, 4kDa MWCO) against PBS. Carboxy-coated microspheres were coupled with capture antibody, using the appropriate coupling buffers. Assays were performed in 96-well filter plates (Millipore Corporation, Billerica, MA) with a vacuum manifold (Millipore) for wash steps and to drain reagents. Incubations were performed at room temperature in the dark on a plate shaker. Serum samples were diluted and added to each well containing beads coupled with the relevant capture antibody. After incubation, plates were washed and the biotinylated detection antibody was added, followed by phycoerythrin-conjugated streptavidin. The median fluorescence intensity (MFI) of approximately 100 microspheres from each sample was analyzed with the Bio-Plex Array reader (Bio-Rad). Eight replicates of an intermediate serum pool made up of 1 part case and 3 parts control serum pools were included on each plate. Readings from patient samples were transformed by dividing by the average MFI of the intermediate pool replicates included on the same plate. Separate experiments showed that this procedure reduces plate-to-plate variation in the results (data not shown).

**Table 7:** Bead-based immunoassay reagents.

Target Protein		Capture Reagents			Detection Reagents		
Gene Symbol	Alias	Antibody	Type	Source	Antibody	Type	Source
MUC16	CA125	anti-CA125 X306	mAb	Research Diagnostics, Inc.	anti-CA125 X52	mAb	Research Diagnostics, Inc.
WFDC2	HE4	anti-HE4 2HS	mAb	Dr. Ingegerd Hellström	anti-HE4 3D8	mAb	Dr. Ingegerd Hellström
MSLN	Mesothelin	anti-MSLN 4H3	mAb	Dr. Ingegerd Hellström	anti-MSLN ovcar569	mAb	Dr. Ingegerd Hellström

The levels of remaining candidate markers in serum or plasma samples were measured by ELISA using kits obtained from commercial suppliers or collaborators (MIF assay was kindly provided by Elliot Segal) according to manufacturers' instructions (Table 6, Table S2). Briefly, freshly thawed serum was diluted with dilution buffer and added to each well. After incubation, plates were washed and incubated with conjugated secondary antibody. Antibody/enzyme conjugates were detected by addition of substrate. Reactions were stopped and optical density was determined using a Spectra Max M2 Microplate Reader (Molecular Devices) at 450 nm or 405 nm with the appropriate baseline correction for each assay. The concentration of each marker was determined using a standard curve that was constructed by plotting the mean optical density obtained for each reference standard provided by the kit against the known concentration. Each sample was tested in duplicate. Laboratories performing the immunoassays were blinded with respect to the case/control status of serum and plasma samples.

### Statistical Methods

Receiver operating characteristic (ROC) curve methods were used to quantify marker performance both graphically and statistically [34]. In order to enable comparison of markers that are measured on different scales, we first transformed all markers (e.g., by their logs) so that the values in the control group appeared normally distributed, and re-scaled so that the Healthy Controls (apparently healthy women followed for at least two years) had a mean of zero and a variance of one [26], [35]. Standardization of the markers does not affect the ROC curves for individual markers but facilitates the comparison of markers because of the uniformity of units of measurement (i.e. the number of standard deviations above the average healthy subject) [26].

Individual markers were ranked by their sensitivity at high specificity in comparing cases to all controls (Healthy Controls, Surgical Benigns and Surgical Normals). We combined markers using approaches that did not require statistical fitting because of the low sample sizes. Application of statistical fitting procedures could have resulted in large biases in biomarker performance. To combine markers, we restricted attention to rules in which elevation of any marker above its respective threshold constitutes a positive result (e.g., an “or” rule). Because all markers were on the same scale, this “or” rule was implemented by using the maximum score of the individual markers in the combined set. For example, consider a patient with normalized MUC16 = 3.67 and normalized WFDC2 = 6.85, meaning that they are 3.67 and 6.85 standard deviations above the mean of the Healthy Controls. The MUC16/WFDC2 combination marker value for this patient is 6.85, the maximum of 3.67 and 6.85. ROC curves may then be calculated from these maximum values. ROC curves, area under the ROC curve (AUC) and partial area under the ROC curve-from specificity = 100% to specificity = 90%- (pAUC) were calculated for all possible combinations of two or three markers. Combination markers were ranked by the estimated sensitivity at 98% specificity. We also reported for each candidate marker a measure of the “discriminatory distance”, which indicated how far the marker levels in the average case were from the average healthy control.

Statistical analyses comparing the pAUC of the most highly ranked markers and combinations of markers were performed using a permutation test [36]. To estimate the distribution of the difference in pAUC under the hypothesis of equality of distribution of the two markers to be tested, we converted data from two markers or combinations into triplicates of the form (Status, Marker 1 value, Marker 2 value). For example, consider an ovarian cancer patient with normalized MUC16 = 3.67 and normalized WFDC2 = 6.85. The corresponding triplicate would be (Case, 3.67, 6.85). Under the null hypothesis of equality of distribution, the two markers were exchangeable. Hence, the permutation distribution was created by choosing a random subset of data points and exchanging Marker 1 value with Marker 2 value. The remaining triplicates were not altered. Using these new markers, we calculated the difference in pAUC (from specificity = 100% to specificity = 90%). We repeated this procedure 1000 times recording the difference in pAUC for each repetition. The reported p-value was the percentage of differences in pAUC under the permutation distribution that were greater than or equal to the observed difference in pAUC of the original markers.

## Supporting Information

Table S1 Detailed assay conditions for in-house bead-based assays for MUC16 (CA125), WFDC2 (HE4) and MSLN (mesothelin).
(0.01 MB XLS)

Table S2 Detailed assay characteristics and conditions for commercial ELISAs.
(0.01 MB XLS)
